# Supplementary material for: Intraindividual epigenetic heterogeneity underlying phenotypic subtypes of advanced prostate cancer
Source: Nat Commun. 2025 Jul 1;16:5543. doi: 10.1038/s41467-025-60654-z (PMC12219151; doi:10.1038/s41467-025-60654-z)
Supplement: Supplementary file 1 — Supplementary Information [file 41467_2025_60654_MOESM1_ESM.pdf]

Supplementary Information

a

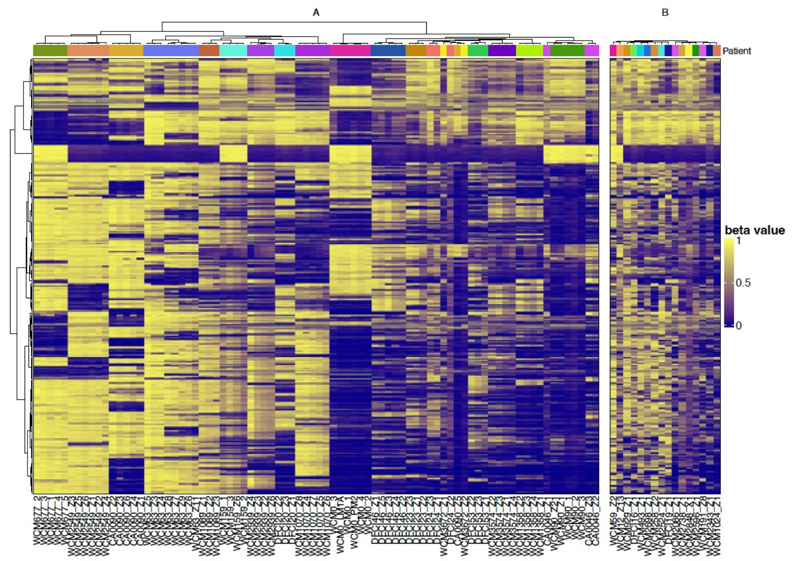

b

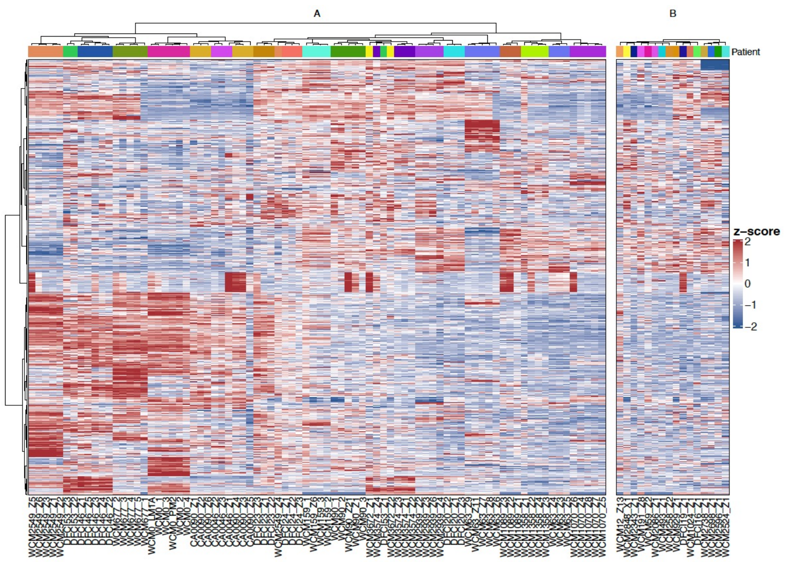

c

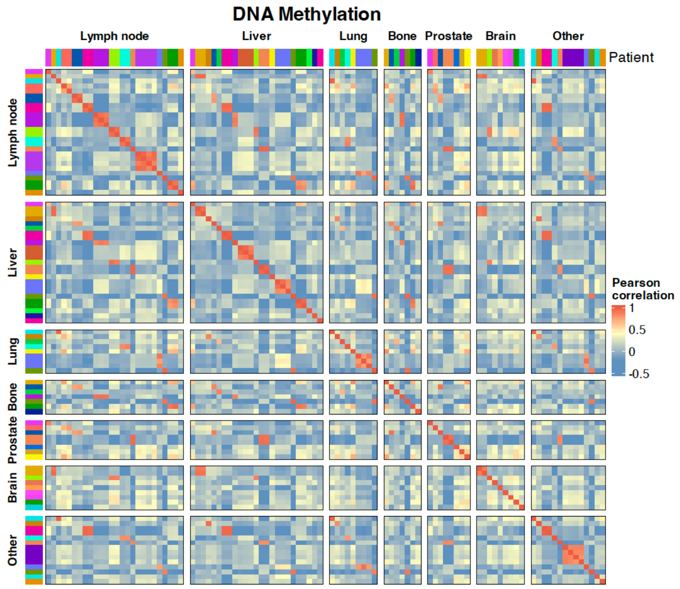

Supplementary Figure 1 | Analysis of DNA methylation and gene expression patterns across samples.

a, Hierarchical clustering (n=98) of DNA methylation profiles using the top 10,000 most variable CpG sites. Panel A (n=82) and B (n=16) show patients with multiple samples and single samples, respectively. Samples are colored by patient ID, with samples from the same patient generally clustering together or appearing adjacent to each other. b, Hierarchical clustering (n=98) of RNA sequencing data using the top 2,000 most variable genes, with panels organized as in (a). Expression patterns, like methylation profiles, are largely preserved within individual patients as shown by the clustering patterns. c, Correlation analysis (n=98) of DNA methylation patterns between samples from the same anatomical site. Results demonstrate that DNA methylation patterns are more strongly influenced by patient-specific factors than anatomical site.

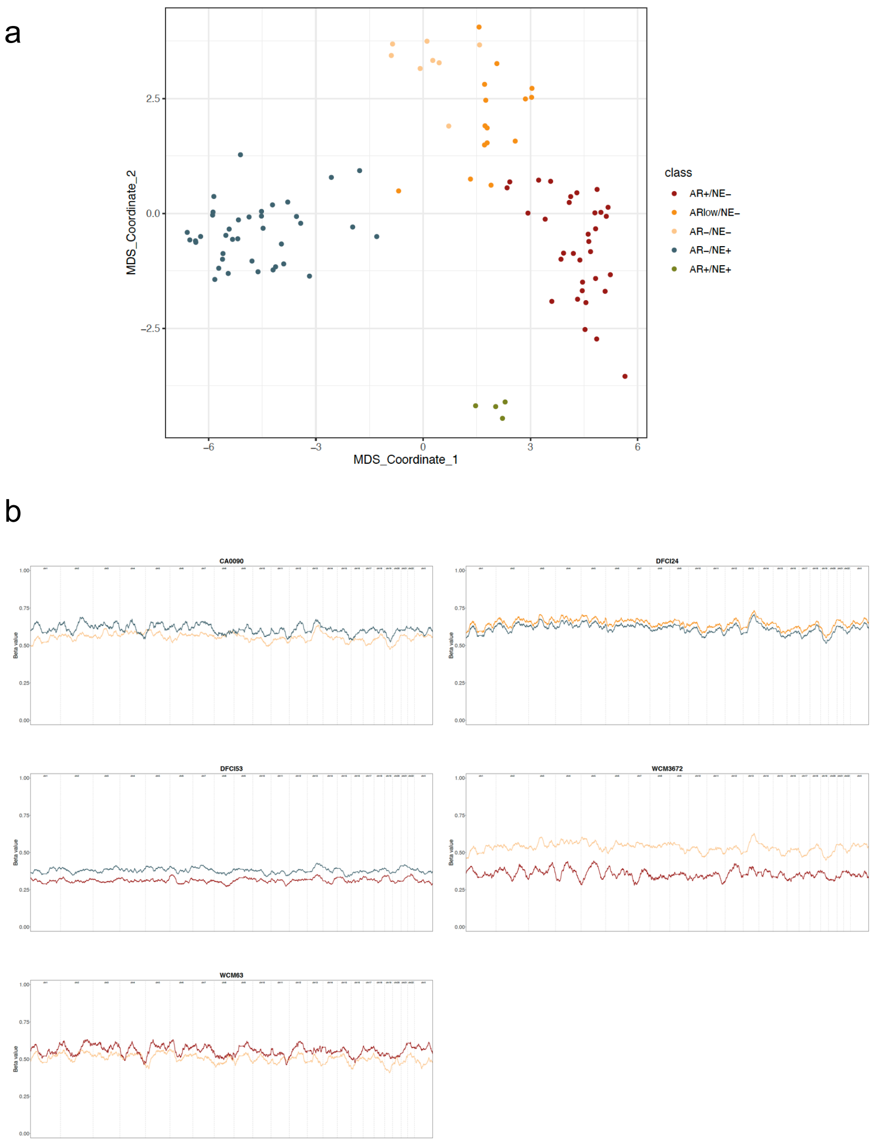

Supplementary Figure 2 | Molecular subtype classification and analysis of cases with intraindividual heterogeneity. a, Multidimensional scaling (MDS) analysis using AR and NE signature gene expression data showing clear separation of molecular subtypes (AR+/NE−, AR-low/NE−, AR−/NE−, AR−/NE+, and AR+/NE+). b, Genome-wide DNA methylation profiles of five cases (CA0090, DFCI24, DFCI53, WCM3672, WCM63) showing intraindividual heterogeneity. Samples are color-coded by molecular subtype, revealing distinct methylation patterns between different molecular subtypes within the same patient. For samples from the same patient with identical molecular subtypes, methylation β values were averaged.

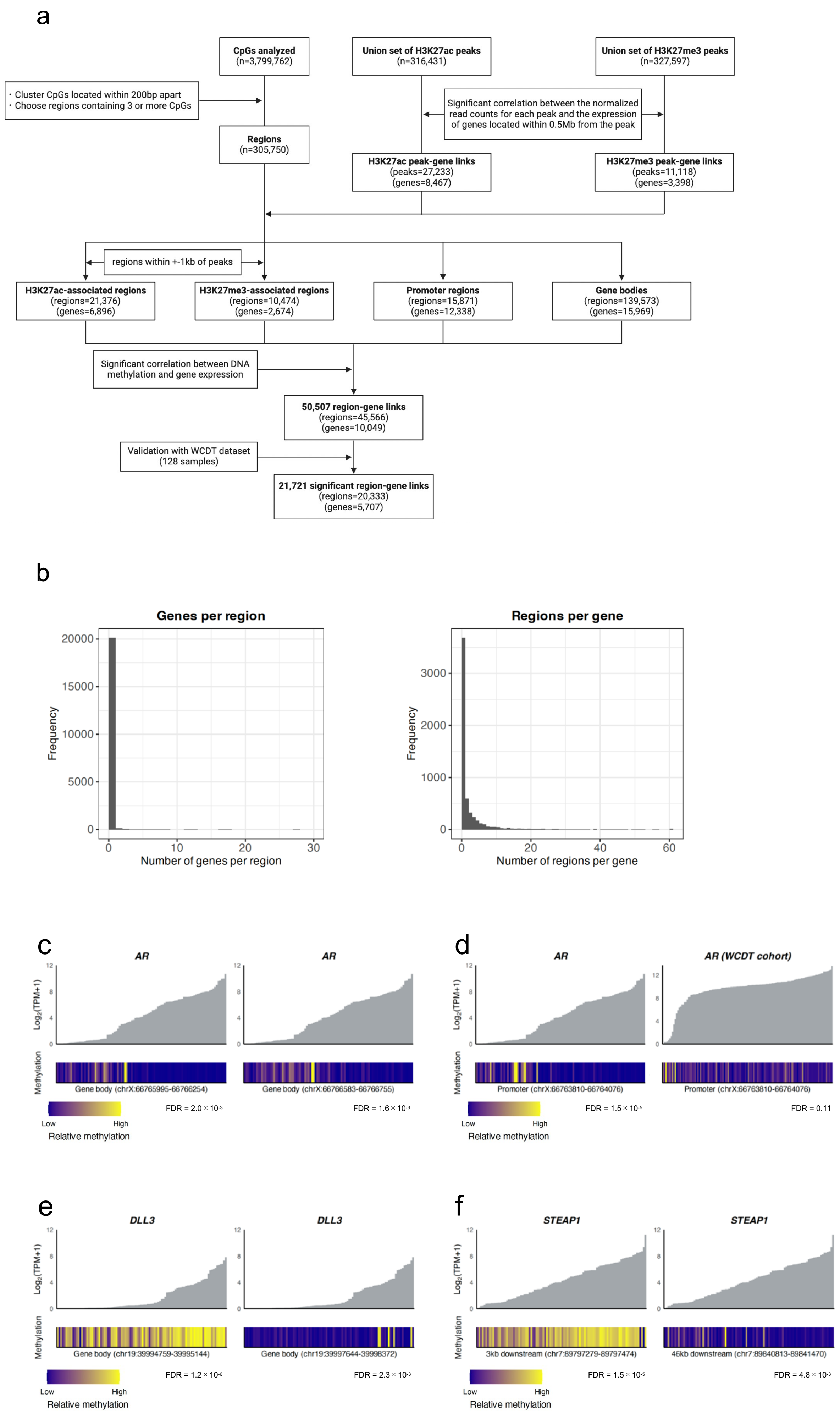
a
CpGs analyzed
(n=3,799,762)
Union set of H3K27ac peaks
(n=316,431)
Union set of H3K27me3 peaks
(n=327,597)
・Cluster CpGs located within 200bp apart
・Choose regions containing 3 or more CpGs
Significant correlation between the normalized read counts for each peak and the expression of genes located within 0.5Mb from the peak
Regions
(n=305,750)
H3K27ac peak-gene links
(peaks=27,233)
(genes=8,467)
H3K27me3 peak-gene links
(peaks=11,118)
(genes=3,398)
regions within +-1kb of peaks
H3K27ac-associated regions
(regions=21,376)
(genes=6,896)
H3K27me3-associated regions
(regions=10,474)
(genes=2,674)
Promoter regions
(regions=15,871)
(genes=12,338)
Gene bodies
(regions=139,573)
(genes=15,969)
Significant correlation between DNA methylation and gene expression
50,507 region-gene links
(regions=45,566)
(genes=10,049)
Validation with WCDT dataset
(128 samples)
21,721 significant region-gene links
(regions=20,333)
(genes=5,707)
b
Genes per region
Frequency
Number of genes per region
Regions per gene
Frequency
Number of regions per gene
c
AR
AR
Log2(TPM+1)
Methylation
Gene body (chrX:66765995-66766254)
Gene body (chrX:66766583-66766755)
FDR = 2.0 × 10-3
FDR = 1.6 × 10-3
Low
High
Relative methylation
d
AR
AR (WCDT cohort)
Promoter (chrX:66763810-66764076)
Promoter (chrX:66763810-66764076)
FDR = 1.5 × 10-5
FDR = 0.11
Relative methylation
e
DLL3
DLL3
Gene body (chr19:39994759-39995144)
Gene body (chr19:39997644-39998372)
FDR = 1.2 × 10-6
FDR = 2.3 × 10-3
Relative methylation
f
STEAP1
STEAP1
3kb downstream (chr7:89797279-89797474)
46kb downstream (chr7:89840813-89841470)
FDR = 1.5 × 10-5
FDR = 4.8 × 10-3
Relative methylation

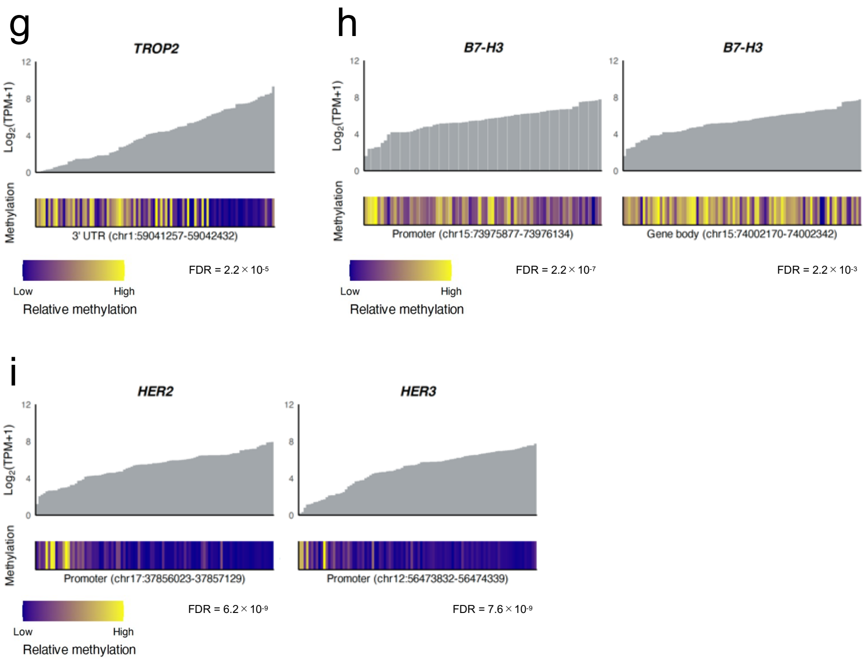

Supplementary Figure 3 | Analysis workflow and DNA methylation-gene expression regulatory chains in AR and therapeutic targets. a, Flow chart showing step-by-step analysis for identification of DNA methylation-gene expression regulatory chains. Each step is annotated with the number of CpG sites, peaks, regions, and/or genes analyzed and retained, from initial data processing through final significant associations. b, Distribution of region-gene links. Left: Distribution of the number of genes linked per region. Right: Distribution of the number of regions linked per gene. c–i, Methylation-expression relationships for AR gene body across samples (n=98) (c), AR promoter in our cohort (n=98) and WCDT cohort[1,2] (n=128) (d), DLL3 (n=98) (e), STEAP1 (n=98) (f), TROP2 (n=98) (g), B7-H3 promoter (n=73, only RRBS samples cover this region) and gene body (n=98) (h), and HER2 (n=98) and HER3 (n=98) (i). Bar plots show sample-level gene expression values, with DNA methylation levels indicated by color. False discovery rate (FDR) values for correlations between gene expression and DNA methylation are shown.

a

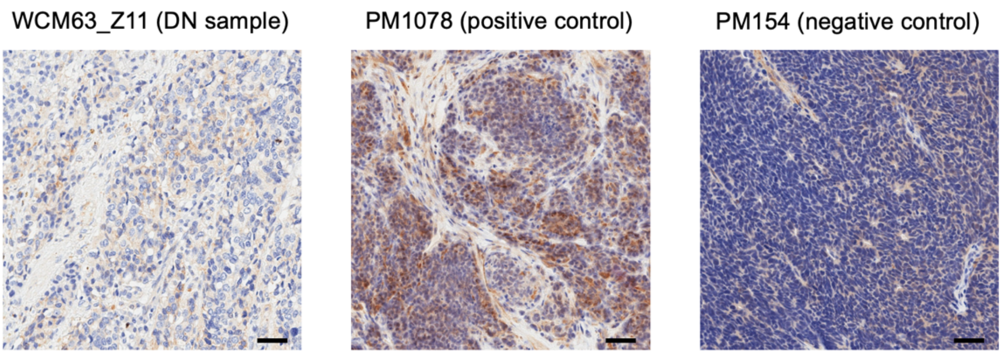
WCM63_Z11 (DN sample)
PM1078 (positive control)
PM154 (negative control)

b

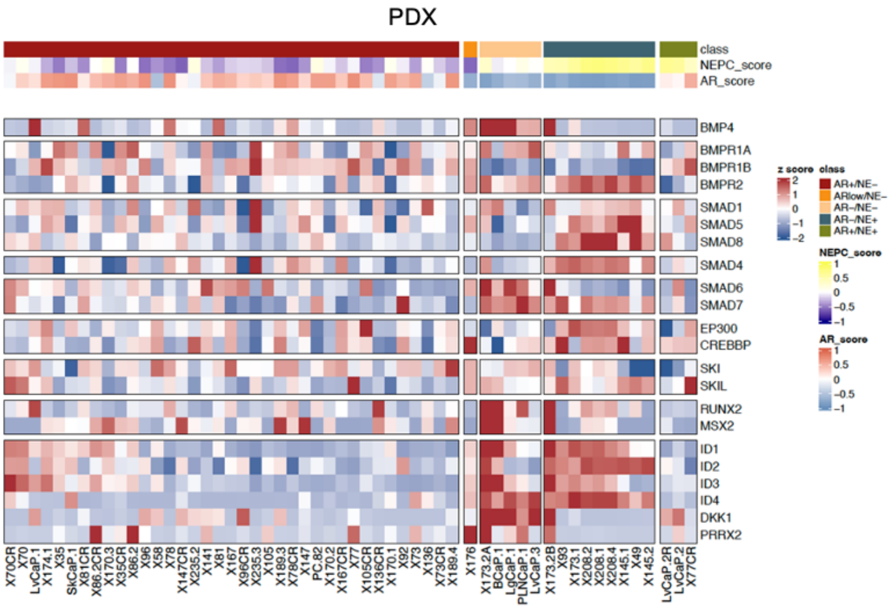
PDX
class
NEPC_score
AR_score
BMP4
BMPR1A
BMPR1B
BMPR2
SMAD1
SMAD5
SMAD8
SMAD4
SMAD6
SMAD7
EP300
CREBBP
SKI
SKIL
RUNX2
MSX2
ID1
ID2
ID3
ID4
DKK1
PRRX2
z score
class
AR+/NE−
ARlow/NE−
AR−/NE−
AR−/NE+
AR+/NE+
NEPC_score
AR_score

c

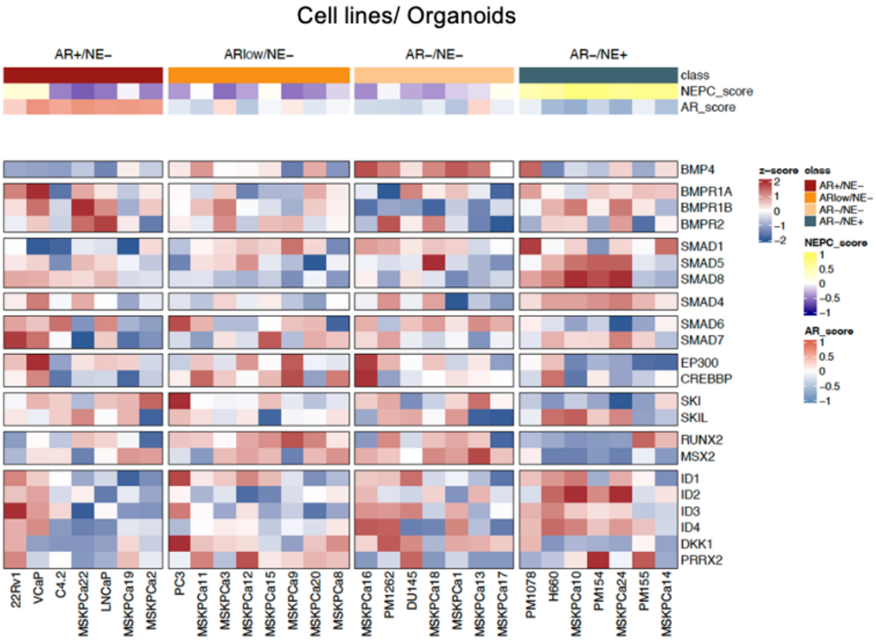
Cell lines/ Organoids
AR+/NE−
ARlow/NE−
AR−/NE−
AR−/NE+
class
NEPC_score
AR_score
BMP4
BMPR1A
BMPR1B
BMPR2
SMAD1
SMAD5
SMAD8
SMAD4
SMAD6
SMAD7
EP300
CREBBP
SKI
SKIL
RUNX2
MSX2
ID1
ID2
ID3
ID4
DKK1
PRRX2
22Rv1
VCaP
C4.2
MSKPCa22
LNCaP
MSKPCa19
MSKPCa2
PC3
MSKPCa11
MSKPCa3
MSKPCa12
MSKPCa15
MSKPCa9
MSKPCa20
MSKPCa8
MSKPCa16
PM1262
DU145
MSKPCa18
MSKPCa1
MSKPCa13
MSKPCa17
PM1078
H660
MSKPCa10
PM154
MSKPCa24
PM155
MSKPCa14
z−score
class
NEPC_score
AR_score

d

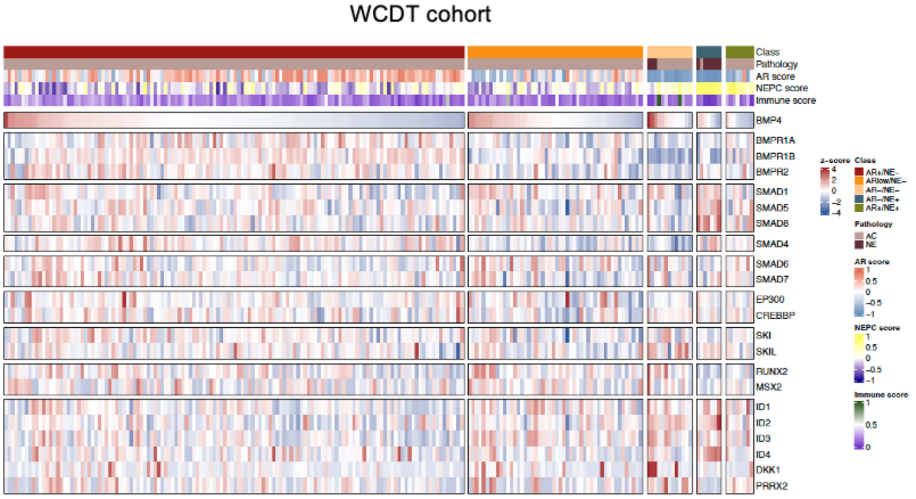
WCDT cohort
Class
Pathology
AR score
NEPC score
Immune score
BMP4
BMPR1A
BMPR1B
BMPR2
SMAD1
SMAD5
SMAD8
SMAD4
SMAD6
SMAD7
EP300
CREBBP
SKI
SKIL
RUNX2
MSX2
ID1
ID2
ID3
ID4
DKK1
PRRX2

e

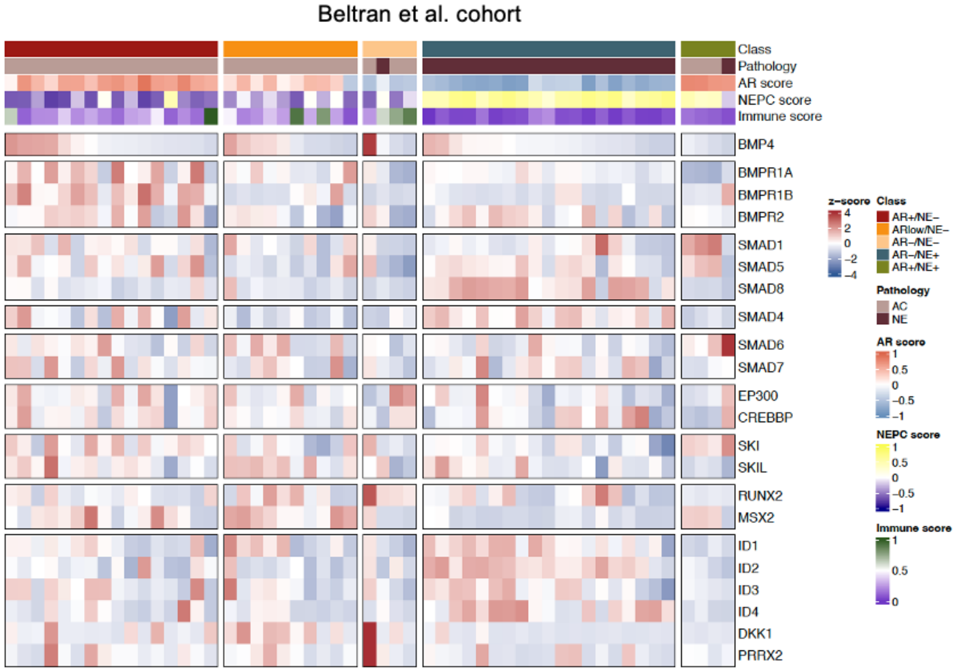
Beltran et al. cohort
Class
Pathology
AR score
NEPC score
Immune score
BMP4
BMPR1A
BMPR1B
BMPR2
SMAD1
SMAD5
SMAD8
SMAD4
SMAD6
SMAD7
EP300
CREBBP
SKI
SKIL
RUNX2
MSX2
ID1
ID2
ID3
ID4
DKK1
PRRX2
z-score
4
2
0
−2
−4
Class
AR+/NE−
ARlow/NE−
AR−/NE−
AR−/NE+
AR+/NE+
Pathology
AC
NE
AR score
1
0.5
0
−0.5
−1
NEPC score
1
0.5
0
−0.5
−1
Immune score
1
0.5
0

f

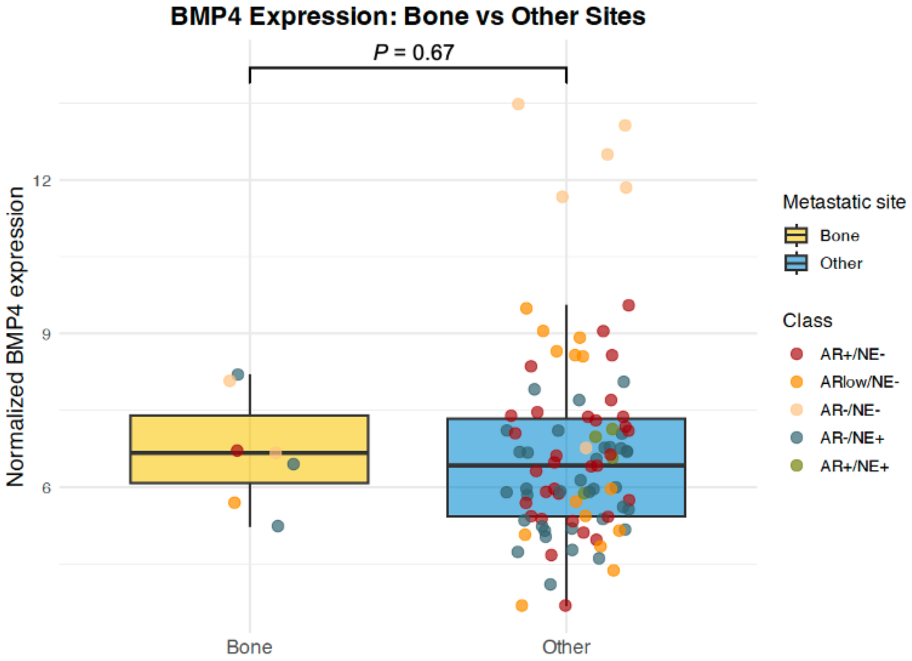
BMP4 Expression: Bone vs Other Sites
P = 0.67
Normalized BMP4 expression
12
9
6
Bone
Other
Metastatic site
Bone
Other
Class
AR+/NE-
ARlow/NE-
AR-/NE-
AR-/NE+
AR+/NE+

g

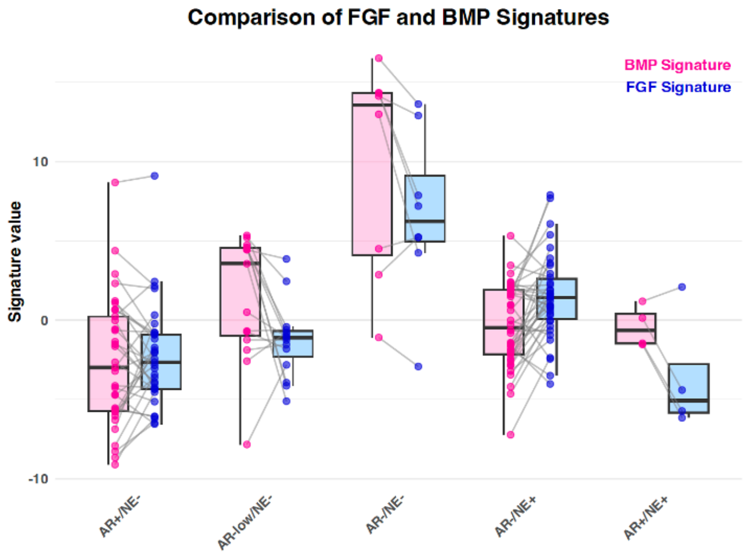

Comparison of FGF and BMP Signatures
BMP Signature
FGF Signature
Signature value
10
0
-10
AR+/NE-
AR-low/NE-
AR-/NE-
AR-/NE+
AR+/NE+

h

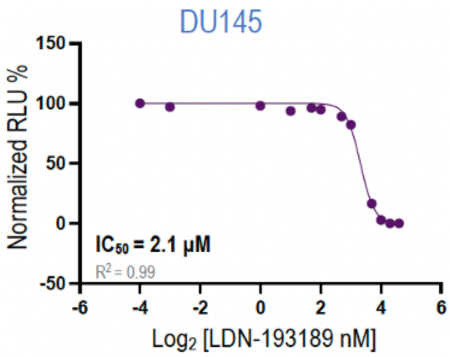

DU145
Normalized RLU %
150
100
50
0
-50
IC50 = 2.1 µM
R2 = 0.99
-6
-4
-2
0
2
4
6
Log2 [LDN-193189 nM]

i

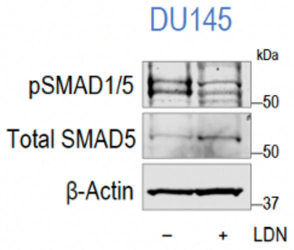

DU145
kDa
pSMAD1/5
Total SMAD5
β-Actin
50
50
37
–
+
LDN

j

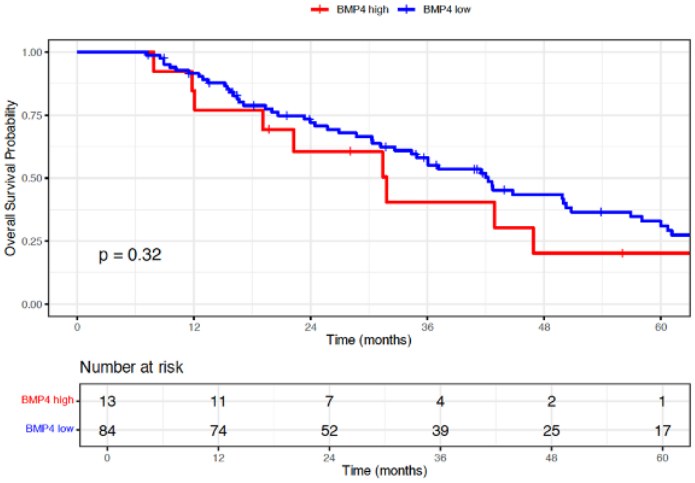

BMP4 high
BMP4 low
Overall Survival Probability
1.00
0.75
0.50
0.25
0.00
p = 0.32
0
12
24
36
48
60
Time (months)
Number at risk
BMP4 high 13 11 7 4 2 1
BMP4 low 84 74 52 39 25 17
Time (months)

k

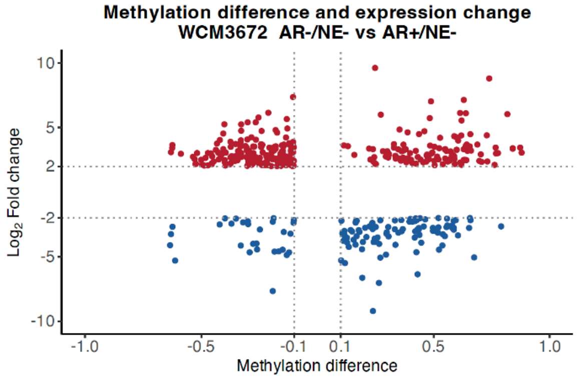

Methylation difference and expression change
WCM3672 AR-/NE- vs AR+/NE-
Log2 Fold change
10
5
2
-2
-5
-10
-1.0
-0.5
-0.1
0.1
0.5
1.0
Methylation difference

l

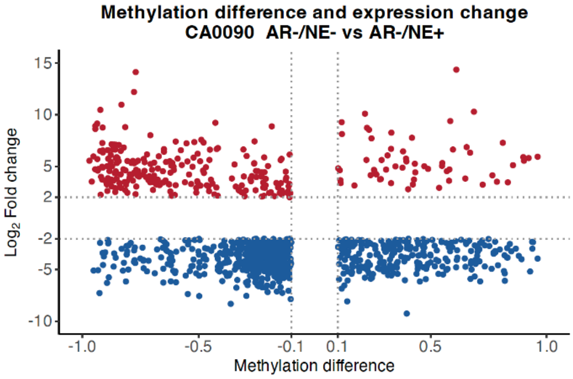

Methylation difference and expression change
CA0090 AR-/NE- vs AR-/NE+
Log2 Fold change
15
10
5
2
-2
-5
-10
-1.0
-0.5
-0.1
0.1
0.5
1.0
Methylation difference

m

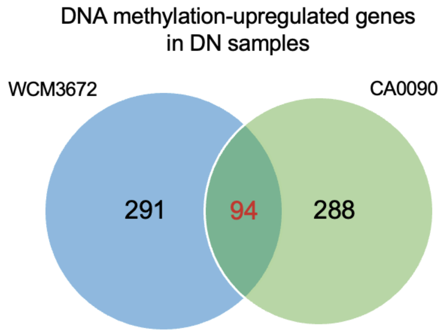

Supplementary Figure 4 | Extended analysis of BMP4 pathway activation and immune features in CRPC subtypes.

a, Immunohistochemical staining of BMP4 in WCM63 double negative sample shows positive expression. PM1078 and PM154 prostate cancer patient-derived organoids serve as positive and negative controls for BMP4 expression, respectively (see Supplementary Fig. 4e for expression levels). Scale bars: 50 μm. b,c, Heatmaps showing expression of BMP4 signaling pathway-related genes in PDX models[3-6] (n=55) (b) and cell lines/organoid models[6] (n=29) (c), with elevated expression in double negative subtypes. d,e, Heatmaps showing expression of BMP4 signaling pathway-related genes in WCDT cohort[1,2] (n=210) (d) and Beltran et al.[7] cohort (n=53) (e). In these patient cohorts, some double negative samples showed high BMP4 pathway activity while others exhibited high immune signatures. f, Analysis of BMP4 expression levels comparing bone metastases (n=7, yellow) and other metastatic sites (n=91, blue), showing no significant difference between these groups. Box plots show median (center line), first and third quartiles (box bounds), and whiskers extending to 1.5× the interquartile range. Individual data points are plotted as overlaid dots, with colors indicating molecular subtypes. Statistical analysis was performed using two-sided Wilcoxon test. g, Comparison of BMP4 (red) and FGF (blue) pathway signature scores across samples, with lines connecting scores from the same sample. Data are grouped by molecular subtypes: AR+/NE− (n=33), AR-low/NE− (n=15), AR−/NE− (n=8), AR−/NE+ (n=38), and AR+/NE+ (n=4). Box plots show median, quartiles and whiskers extending to 1.5× the interquartile range. Individual data points are plotted as overlaid dots. h, DU145 cells (n=5/condition) treated with increasing doses of LDN-193189 (LDN, ALK inhibitor) for 6 days and CellTiter-Glo® luminescent cell viability assay was performed and IC50 was measured. Dots, means of values (n=5/condition); line, four-parameter logistic curve. i, Western blot analysis in DNPC DU145 cell line confirming reduction in phospho-SMAD1/5 upon 2 μM LDN-193189 treatment for 6 days. Blots were reprobed for total-SMAD5 and β-actin as control. Representative western blot from two independent experiments is shown. j, Kaplan-Meier analysis of overall survival in the WCDT mCRPC cohort[1,2] stratified by BMP4 expression. Patients were categorized into high expression (red line, n=13) and low expression (blue line, n=84) groups based on median BMP4 expression. Numbers at risk are shown below the graph. Statistical significance was assessed using the log-rank test (p=0.32). k, DNA methylation-regulated genes identified in WCM3672 AR−/NE− versus AR+/NE− samples. Red and blue dots indicate highly upregulated and downregulated genes, respectively, that show significant expression changes and substantial methylation differences in their associated regions. l, DNA methylation-regulated genes identified in CA0090 AR−/NE− versus AR−/NE+ samples, displayed as in (k). m, Venn diagram showing the overlap of DNA methylation-upregulated genes in two immune-high DN CRPC samples. WCM3672 sample contained 291 methylation-upregulated genes and CA0090 sample contained 288 such genes, with 94 genes commonly upregulated in both samples.

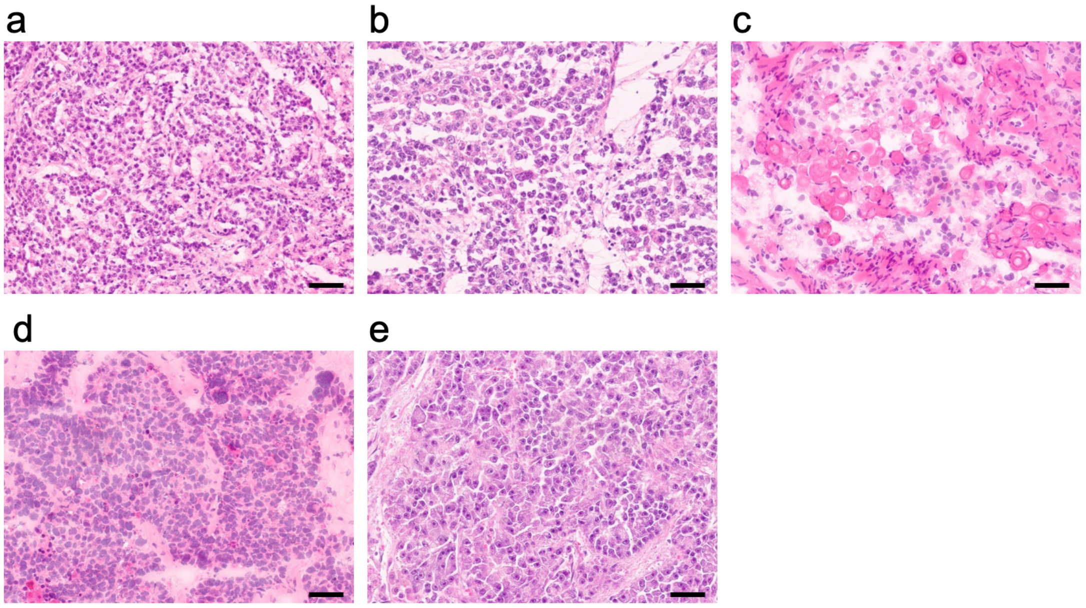

Supplementary Figure 5 | Representative histological images from different molecular subtypes of metastatic CRPC. Representative hematoxylin and eosin (H&E) stained sections from tumor samples classified according to molecular subtyping. a, AR+/NE－. b, AR-low/NE－. c, AR－/NE－ (double negative). d, AR－/NE+. e, AR+/NE+. These histological images illustrate the morphological features of samples analyzed in this study. Scale bars: 50 μm.

Patient ID	Age at diagnosis	No. of prior systemic therapies	Prior systemic therapies	PSA at Autopsy/Biopsy (ng/ml)	Pathology	Site of mets	Autopsy (Y/N)
CA0046	50–59	2	ADT, CBDCA+ETP	0.1	NE	Liver, LN	Y
CA0090	60–69	2	ADT, CBDCA+ETP	0.1	NE	Brain, Liver, Bone, LN	Y
DFCI16	60–69	5	ADT, ABI, ENZ, DTX, radium-223	49.8	AC	Lung, Liver, Bone, LN	Y
DFCI19	60–69	7	ADT, ENZ, ABI, DTX, olaparib, pembrolizumab, LuPSMA	267	AC	Lung, Liver, LN, Abdominal mass	Y
DFCI20	60–69	6	ADT, ABI, ENZ, radium-223, DTX, CBZ	2618	AC	Lung, Bone, LN, Pleura	Y
DFCI23	50–59	4	ADT, ENZ, CBDCA+ETP, lurbinectedin	22.9	NE	Lung, Liver, LN	Y
DFCI24	50–59	3	ADT, ENZ, CBDCA	50	NE	Lung, Liver, LN	Y
DFCI46	50–59	2	ADT, ABI	0.07	NE	Liver, Bone, LN	Y
DFCI53	70–79	5	ADT, DTX, CBZ, darolutamide, LuPSMA	5.67	AC	Lung, Liver, Bone, LN	Y
WCM0	50–59	1	CBDCA+ETP	0.03	NE	Liver, LN, Pelvic mass	Y
WCM12	60–69	3	ADT, DTX, CBDCA+ETP	0.01	NE	Brain	N
WCM59	70–79	NA	NA	76.2	AC	Liver	N
WCM63	50–59	8	ADT, BIC, sipuleucel T, DTX, ENZ, radium-223, ABI, CBDCA+CBZ	1.03	AC	Lung, Liver, LN, Diaphragm	Y
WCM90	50–59	4	ADT, BIC, DTX, ABI	22.25	AC	Liver, Bone, LN	Y
WCM159	60–69	4	ADT, BIC, DTX, ABI	1612	AC	Lung, LN, Adrenal gland	Y
WCM191	50–59	9	ADT, flutamide, nilutamide, sipuleucel T, ENZ, orteronel, DTX, ABI, radium-223	153.62	AC	Brain	N
WCM258	50–59	8	ADT, ENZ, ABI, DTX, CBZ, CBDCA, olaparib, pembrolizumab	5.1	AC	LN	N
WCM493	50–59	4	ADT, DTX, sipuleucel T, ENZ	45.36	AC	Brain	N
WCM677	NA	2	ADT, radium-223	0.32	NE	Lung, Liver, Bone, LN, Peritoneum	Y
WCM929	60–69	5	ADT, BIC, nilutamide, ABI, ENZ	39.79	AC	Bladder	N
WCM1024	40–49	6	ADT, sipuleucel T, ENZ, ABI, CBDCA+PTX, vinorelbine	6.81	AC	Brain	N
WCM1070	50–59	4	ADT, DTX, sipuleucel T, ENZ	5.86	AC	Liver, Bone, LN	Y
WCM1089	60–69	6	ADT, BIC, sipuleucel T, DTX, ABI, radium-223	7.17	AC	Liver	Y
WCM1358	40–49	6	ADT, BIC, sipuleucel T, ABI, DTX, CBDCA+CBZ	110.24	AC	Brain, Liver, LN	Y
WCM2086	50–59	4	ADT, ENZ, radium-223, DTX	549.9	AC	Brain, Bone	N
WCM2343	70–79	4	ADT, ENZ, ABI, CBDCA+CBZ	0.05	AC	Lung, Bone, LN	N
WCM2524	70–79	2	ADT, dexamethasone	723.19	AC	Bone	N
WCM2549	70–79	2	CDDP+ETP, talabostat+pembrolizumab	20.05	NE	Liver, Bone, LN, Adrenal gland	Y
WCM2596	60–69	4	ADT, ENZ, bavdegalutamide, radium-223	776.67	AC	Brain, Bone, LN	N
WCM2698	60–69	1	ADT	24.47	AC	LN	N
WCM2738	60–69	NA	NA	6.1	AC	None	N
WCM2848	60–69	2	ADT, CBDCA+CBZ	0.3	AC	Bone, LN	N
WCM2939	60–69	3	ADT, ENZ, sipuleucel T	281.72	AC	Bone, LN	Y
WCM3574	50–59	2	ADT, CBZ	NA	AC	Brain, Bone, Bladder, Kidney, Pancreas	Y
WCM3672	50–59	3	ADT, ABI, DTX	18.11	AC	Lung, Liver, Bone, LN	Y

Abbreviations: ABI, abiraterone; ADT, androgen deprivation therapy; BIC, bicalutamide; CBDCA, carboplatin; CBZ, cabazitaxel; DTX, docetaxel; ENZ, enzalutamide; ETP, etoposide; LuPSMA, 177Lu-labeled prostate-specific membrane antigen; PTX, paclitaxel; AC, adenocarcinoma; NE, neuroendocrine carcinoma; LN, lymph node; NA, not available.

Supplementary Table 1 | Clinical characteristics of patients analyzed

ID	Description	Count	p.adjust	Genes
GO:0042476	odontogenesis	22	7.50E-11	*BMP4* , *DLX3* , *LEF1* , *MSX2* , *RUNX2* , etc.
GO:0001763	morphogenesis of a branching structure	26	1.03E-10	*BMP4* , *LEF1* , *MSX2* , *WNT5A* , etc.
GO:0048736	appendage development	23	2.01E-09	*BMP4* , *LEF1* , *MSX2* , *RUNX2* , *WNT5A* , etc.
GO:0060173	limb development	23	2.01E-09	*BMP4* , *LEF1* , *MSX2* , *RUNX2* , *WNT5A* , etc.
GO:0042475	odontogenesis of dentin-containing tooth	17	3.07E-09	*BMP4* , *DLX3* , *LEF1* , *MSX2* , *RUNX2* , etc.
GO:0048754	branching morphogenesis of an epithelial tube	21	3.62E-09	*BMP4* , *LEF1* , *MSX2* , *WNT5A* , etc.
GO:2000027	regulation of animal organ morphogenesis	18	2.65E-08	*BMP4* , *RUNX2* , *WNT5A* , etc.

Supplementary Table 2 | GO terms enriched with *BMP4* and its associated genes in WCM63 double negative tumors.

Seven GO terms among the top 10 enriched terms from methylation-upregulated genes containing *BMP4* and BMP4 signaling pathway-related genes as shared genes. Statistical analysis for GO enrichment was performed using one-sided Fisher's exact test with Benjamini-Hochberg correction for multiple testing.

ID	Description	Count	p.adjust	Genes
GO:0007159	leukocyte cell-cell adhesion	23	2.86E-15	*VAV1* , *L4R* , *CD300A* , *IITGB2* , *CARD11*
GO:0002768	immune response-regulating cell surface receptor signaling pathway	21	1.54E-14	*VAV1* , *CD300A* , *CARD11*
GO:0002429	immune response-activating cell surface receptor signaling pathway	18	5.86E-12	*VAV1* , *CD300A* , *CARD11*
GO:1903037	regulation of leukocyte cell-cell adhesion	19	5.86E-12	*VAV1* , *IL4R* , *CD300A* , *ITGB2* , *CARD11*
GO:0030098	lymphocyte differentiation	19	2.09E-11	*VAV1* , *IL4R* , *CARD11*
GO:0043299	leukocyte degranulation	11	2.68E-11	*IL4R* , *ITGB2*
GO:1903039	positive regulation of leukocyte cell-cell adhesion	16	5.04E-11	*VAV1* , *IL4R* , *ITGB2* , *CARD11*
GO:0002274	myeloid leukocyte activation	15	8.96E-11	*IL4R* , *ITGB2*
GO:0002443	leukocyte mediated immunity	19	1.17E-10	*VAV1* , *IL4R* , *ITGB2*
GO:0030217	T cell differentiation	16	2.20E-10	*VAV1* , *IL4R* , *CARD11*

Supplementary Table 3 | GO analysis results of commonly methylation-upregulated genes in immune-high DN CRPC samples.

Top 10 immune-related GO terms enriched in the analysis of commonly methylation-upregulated genes, highlighting shared immune-regulatory genes between immune-high DN CRPC samples. Statistical analysis for GO enrichment was performed using one-sided Fisher's exact test with Benjamini-Hochberg correction for multiple testing.

Supplementary References

1. Zhao, S.G. *et al.* The DNA methylation landscape of advanced prostate cancer. *Nat Genet* **52**, 778-789 (2020).
2. Lundberg, A. *et al.* The Genomic and Epigenomic Landscape of Double-Negative Metastatic Prostate Cancer. *Cancer Res* **83**, 2763-2774 (2023).
3. Labrecque, M.P. *et al.* Molecular profiling stratifies diverse phenotypes of treatment-refractory metastatic castration-resistant prostate cancer. *J Clin Invest* **129**, 4492-4505 (2019).
4. Brennen, W.N. *et al.* Resistance to androgen receptor signaling inhibition does not necessitate development of neuroendocrine prostate cancer. *JCI Insight* **6**, e146827 (2021).
5. Coleman, I.M. *et al.* Therapeutic Implications for Intrinsic Phenotype Classification of Metastatic Castration-Resistant Prostate Cancer. *Clin Cancer Res* **28**, 3127-3140 (2022).
6. Tang, F. *et al.* Chromatin profiles classify castration-resistant prostate cancers suggesting therapeutic targets. *Science* **376**, eabe1505 (2022).
7. Beltran, H. *et al.* Divergent clonal evolution of castration-resistant neuroendocrine prostate cancer. *Nat Med* **22**, 298-305 (2016).
