# Supplementary material for: Intraindividual epigenetic heterogeneity underlying phenotypic subtypes of advanced prostate cancer
Source: Nat Commun. 2025 Jul 1;16:5543. doi: 10.1038/s41467-025-60654-z (PMC12219151; doi:10.1038/s41467-025-60654-z)
Supplement: Supplementary file 2 — Reporting Summary [file 41467_2025_60654_MOESM2_ESM.pdf]

nature portfolio

Corresponding author(s): Dr. Himisha Beltran

Last updated by author(s): May 22, 2025

Reporting Summary

Nature Portfolio wishes to improve the reproducibility of the work that we publish. This form provides structure for consistency and transparency in reporting. For further information on Nature Portfolio policies, see our Editorial Policies and the Editorial Policy Checklist.

Statistics

For all statistical analyses, confirm that the following items are present in the figure legend, table legend, main text, or Methods section.

n/a	Confirmed	
☐	☒	The exact sample size (*n*) for each experimental group/condition, given as a discrete number and unit of measurement
☐	☒	A statement on whether measurements were taken from distinct samples or whether the same sample was measured repeatedly
☐	☒	The statistical test(s) used AND whether they are one- or two-sided<br>*Only common tests should be described solely by name; describe more complex techniques in the Methods section.*
☐	☒	A description of all covariates tested
☐	☒	A description of any assumptions or corrections, such as tests of normality and adjustment for multiple comparisons
☐	☒	A full description of the statistical parameters including central tendency (e.g. means) or other basic estimates (e.g. regression coefficient) AND variation (e.g. standard deviation) or associated estimates of uncertainty (e.g. confidence intervals)
☐	☒	For null hypothesis testing, the test statistic (e.g. *F*, *t*, *r*) with confidence intervals, effect sizes, degrees of freedom and *P* value noted<br>*Give P values as exact values whenever suitable.*
☒	☐	For Bayesian analysis, information on the choice of priors and Markov chain Monte Carlo settings
☒	☐	For hierarchical and complex designs, identification of the appropriate level for tests and full reporting of outcomes
☐	☒	Estimates of effect sizes (e.g. Cohen's *d*, Pearson's *r*), indicating how they were calculated

Our web collection on statistics for biologists contains articles on many of the points above.

Software and code

Policy information about availability of computer code

Data collection	Sequencing data was collected using standard commercially available sequencers (Illumina NovaSeq 6000).
Data analysis	For RRBS data, raw sequencing data were processed using the Bismark pipeline, which encompassed quality control, alignment with the GRCh37 reference genome, and methylation calling. For RNA-seq data, raw sequencing data were then processed using the STAR aligner for mapping to the human reference genome, followed by quantification of gene expression using RSEM. Read counts were normalized using DESeq2. For ChIP-seq data, sequenced reads were mapped to GRCh37 using bowtie2 and the data were processed using the MACS2 pipeline for peak calling. We used the "-f BAM" flag with a genome size of 2.7e9 and the standard FDR threshold of 0.01. For H3K27me3 data, the flag "--broad" was used. For CUT&Tag data, sequenced reads were mapped to GRCh37 using bowtie2 and the data were processed using the MACS2 pipeline for peak calling. We used the "-f BAMPE" flag with a genome size of 2.7e9 and the standard FDR threshold of 0.01. For H3K27me3 data, the flag "--broad" was used. A union set of peaks was generated using BEDTools. For each sample, fragment counts within each histone peak were extracted using the getCounts function from chromVAR, followed by variance-stabilizing transformation (vst) normalization using DESeq2.

For manuscripts utilizing custom algorithms or software that are central to the research but not yet described in published literature, software must be made available to editors and reviewers. We strongly encourage code deposition in a community repository (e.g. GitHub). See the Nature Portfolio guidelines for submitting code & software for further information.

Data

Policy information about availability of data

All manuscripts must include a data availability statement. This statement should provide the following information, where applicable:

- Accession codes, unique identifiers, or web links for publicly available datasets
- A description of any restrictions on data availability
- For clinical datasets or third party data, please ensure that the statement adheres to our policy

RRBS, RNA-seq, ChIP-seq and CUT&Tag for H3K27ac and H3K27me3 data of patient tumor samples generated in this study have been deposited in the public repository (Gene Expression Omnibus) under accession codes GSE289466 [https://www.ncbi.nlm.nih.gov/geo/query/acc.cgi?acc=GSE289466], GSE289467 [https://www.ncbi.nlm.nih.gov/geo/query/acc.cgi?acc=GSE289467], GSE289468 [https://www.ncbi.nlm.nih.gov/geo/query/acc.cgi?acc=GSE289468], and GSE289605 [https://www.ncbi.nlm.nih.gov/geo/query/acc.cgi?acc=GSE289605]. The RNA-seq data of mCRPC tumors from the WCDT cohort are available in the European Genome-Phenome Archive database under accession codes EGAD00001008991 [https://ega-archive.org/datasets/EGAD00001008991], EGAD00001008487 [https://www.ega-archive.org/datasets/EGAD00001008487], and EGAD00001009065 [https://www.ega-archive.org/datasets/EGAD00001009065]5,6. The publicly available RNA-seq data of LuCaP and other PDX models, cell lines, and organoids are available in the Gene Expression Omnibus database under accession codes GSE126078 [https://www.ncbi.nlm.nih.gov/geo/query/acc.cgi?acc=GSE126078]15, GSE160393 [https://www.ncbi.nlm.nih.gov/geo/query/acc.cgi?acc=GSE160393] 27, GSE199596 [https://www.ncbi.nlm.nih.gov/geo/query/acc.cgi?acc=GSE199596]28, and GSE199190 [https://www.ncbi.nlm.nih.gov/geo/query/acc.cgi?acc=GSE199190]8. The publicly available methylation data of mCRPC tumors from the WCDT cohort are available in the dbGaP database under accession codes phs001648 [https://www.ncbi.nlm.nih.gov/projects/gap/cgi-bin/study.cgi?study_id=phs001648.v2.p1]5 and in the European Genome-Phenome Archive database under accession codes EGAS00001006649 [https://ega-archive.org/studies/EGAS00001006649]5,6. The methylation data from LuCaP PDX models are available in the Gene Expression Omnibus database under accession codes GSE205056 [https://www.ncbi.nlm.nih.gov/geo/query/acc.cgi?acc=GSE227853]54. All raw data used to generate the figures presented in this manuscript are available in the Source Data file. Source data are provided with this paper.

Research involving human participants, their data, or biological material

Policy information about studies with human participants or human data. See also policy information about sex, gender (identity/presentation), and sexual orientation and race, ethnicity and racism.

Reporting on sex and gender	Male.
Reporting on race, ethnicity, or other socially relevant groupings	NA
Population characteristics	NA
Recruitment	NA
Ethics oversight	NA

Note that full information on the approval of the study protocol must also be provided in the manuscript.

Field-specific reporting

Please select the one below that is the best fit for your research. If you are not sure, read the appropriate sections before making your selection.

☒ Life sciences ☐ Behavioural & social sciences ☐ Ecological, evolutionary & environmental sciences

For a reference copy of the document with all sections, see nature.com/documents/nr-reporting-summary-flat.pdf

Life sciences study design

All studies must disclose on these points even when the disclosure is negative.

Sample size	Sample sizes are described in the methods and/or figures legends.
Data exclusions	No data was excluded.
Replication	Due to the use of patient samples, replication is not possible.
Randomization	Based on DNA methylation and gene expression patterns in patient samples, we performed clustering analysis and conducted comparisons both between and within groups. No randomization was performed.
Blinding	Since this study used patient samples, blinding was not possible.

Reporting for specific materials, systems and methods

We require information from authors about some types of materials, experimental systems and methods used in many studies. Here, indicate whether each material, system or method listed is relevant to your study. If you are not sure if a list item applies to your research, read the appropriate section before selecting a response.

Materials & experimental systems

n/a	Involved in the study
☐	☒ Antibodies
☐	☒ Eukaryotic cell lines
☒	☐ Palaeontology and archaeology
☒	☐ Animals and other organisms
☒	☐ Clinical data
☒	☐ Dual use research of concern
☒	☐ Plants

Methods

n/a	Involved in the study
☐	☒ ChIP-seq
☒	☐ Flow cytometry
☒	☐ MRI-based neuroimaging

Antibodies

Antibodies used: Primary antibodies against H3K27me3 (cell signaling technology; cat. no. 9733S; 1:2000 dilution) or H3K27ac (Diagenode; cat. no. C15410196; 1:2000 dilution), or BMP4 (antibodies.com; cat. no. A80532; 1:50 dilution) were used.

Validation: All antibodies were purchased after verifying appropriate validation presented in the vendor's website.

Eukaryotic cell lines

Policy information about cell lines and Sex and Gender in Research

Cell line source(s): DU145: Catalog number: HTB-81, ATCC (Manassas, VA)

Authentication: STR profiling

Mycoplasma contamination: DU145 cell line was negative for mycoplasma contamination.

Commonly misidentified lines (See ICLAC register): None

Plants

Seed stocks: *Report on the source of all seed stocks or other plant material used. If applicable, state the seed stock centre and catalogue number. If plant specimens were collected from the field, describe the collection location, date and sampling procedures.*

Novel plant genotypes: *Describe the methods by which all novel plant genotypes were produced. This includes those generated by transgenic approaches, gene editing, chemical/radiation-based mutagenesis and hybridization. For transgenic lines, describe the transformation method, the number of independent lines analyzed and the generation upon which experiments were performed. For gene-edited lines, describe the editor used, the endogenous sequence targeted for editing, the targeting guide RNA sequence (if applicable) and how the editor was applied.*

Authentication: *Describe any authentication procedures for each seed stock used or novel genotype generated. Describe any experiments used to assess the effect of a mutation and, where applicable, how potential secondary effects (e.g. second site T-DNA insertions, mosiacism, off-target gene editing) were examined.*

ChIP-seq

Data deposition

☒ Confirm that both raw and final processed data have been deposited in a public database such as GEO.

☒ Confirm that you have deposited or provided access to graph files (e.g. BED files) for the called peaks.

Data access links
May remain private before publication.

The record GSE289467 is currently private and can be viewed using the reviewer access code: wxyrawyynbgzjkb
The record GSE289468 is currently private and can be viewed using the reviewer access code: exstwkkejvadjcn

Files in database submission

GSM8791938 DFCI23_Z3, H3K27ac, CUT&Tag
GSM8791939 DFCI23_Z3, H3K27me3, CUT&Tag
GSM8791940 DFCI24_Z2, H3K27ac, CUT&Tag
GSM8791941 DFCI24_Z2, H3K27me3, CUT&Tag
GSM8791942 DFCI24_Z3, H3K27ac, CUT&Tag
GSM8791943 WCM1070_Z4, H3K27ac, CUT&Tag
GSM8791944 WCM1070_Z4, H3K27me3, CUT&Tag
GSM8791945 WCM1070_Z5, H3K27ac, CUT&Tag

GSM8791946 WCM1070_Z5, H3K27me3, CUT&Tag
GSM8791947 WCM1070_Z6, H3K27ac, CUT&Tag
GSM8791948 WCM1070_Z6, H3K27me3, CUT&Tag
GSM8791949 WCM1070_Z7, H3K27ac, CUT&Tag
GSM8791950 WCM1070_Z7, H3K27me3, CUT&Tag
GSM8791951 WCM1070_Z8, H3K27ac, CUT&Tag
GSM8791952 WCM1070_Z8, H3K27me3, CUT&Tag
GSM8791953 WCM1358_Z2, H3K27ac, CUT&Tag
GSM8791954 WCM1358_Z2, H3K27me3, CUT&Tag
GSM8791955 WCM1358_Z3, H3K27ac, CUT&Tag
GSM8791956 WCM1358_Z3, H3K27me3, CUT&Tag
GSM8791957 WCM2549_Z1, H3K27ac, CUT&Tag
GSM8791958 WCM2549_Z1, H3K27me3, CUT&Tag
GSM8791959 WCM2549_Z2, H3K27ac, CUT&Tag
GSM8791960 WCM2549_Z2, H3K27me3, CUT&Tag
GSM8791961 WCM2549_Z3, H3K27ac, CUT&Tag
GSM8791962 WCM2549_Z3, H3K27me3, CUT&Tag
GSM8791963 WCM2549_Z4, H3K27ac, CUT&Tag
GSM8791964 WCM2549_Z4, H3K27me3, CUT&Tag
GSM8791965 WCM2549_Z5, H3K27ac, CUT&Tag
GSM8791966 WCM2549_Z5, H3K27me3, CUT&Tag
GSM8791967 WCM2549_Z6, H3K27ac, CUT&Tag
GSM8791968 WCM2549_Z6, H3K27me3, CUT&Tag
GSM8791969 WCM2848_X1, H3K27me3, CUT&Tag
GSM8791970 WCM493_Z1, H3K27ac, CUT&Tag
GSM8791971 WCM493_Z1, H3K27me3, CUT&Tag
GSM8791972 WCM63_Z11, H3K27ac, CUT&Tag
GSM8791973 WCM63_Z11, H3K27me3, CUT&Tag
GSM8791974 WCM63_Z3, H3K27ac, CUT&Tag
GSM8791975 WCM63_Z3, H3K27me3, CUT&Tag
GSM8791976 WCM63_Z4, H3K27ac, CUT&Tag
GSM8791977 WCM63_Z4, H3K27me3, CUT&Tag
GSM8791978 WCM63_Z5, H3K27ac, CUT&Tag
GSM8791979 WCM63_Z5, H3K27me3, CUT&Tag
GSM8791980 WCM63_Z6, H3K27ac, CUT&Tag
GSM8791981 WCM63_Z6, H3K27me3, CUT&Tag
GSM8791982 WCM63_Z7, H3K27ac, CUT&Tag
GSM8791983 WCM63_Z7, H3K27me3, CUT&Tag
GSM8791984 WCM63_Z8, H3K27ac, CUT&Tag
GSM8791985 WCM63_Z8, H3K27me3, CUT&Tag
GSM8791986 WCM63_Z9, H3K27ac, CUT&Tag
GSM8791987 WCM63_Z9, H3K27me3, CUT&Tag
GSM8791988 WCM0_2, H3K27ac, ChIP-seq
GSM8791989 WCM0_2, H3K27me3, ChIP-seq
GSM8791990 WCM0_3, H3K27ac, ChIP-seq
GSM8791991 WCM0_3, H3K27me3, ChIP-seq
GSM8791992 WCM0_4, H3K27ac, ChIP-seq
GSM8791993 WCM0_4, H3K27me3, ChIP-seq
GSM8791994 WCM12_Z13, H3K27ac, ChIP-seq
GSM8791995 WCM12_Z13, H3K27me3, ChIP-seq
GSM8791996 WCM159_1, H3K27ac, ChIP-seq
GSM8791997 WCM159_1, H3K27me3, ChIP-seq
GSM8791998 WCM159_2, H3K27ac, ChIP-seq
GSM8791999 WCM159_2, H3K27me3, ChIP-seq
GSM8792000 WCM159_Z6, H3K27ac, ChIP-seq
GSM8792001 WCM159_Z6, H3K27me3, ChIP-seq
GSM8792002 WCM677_1, H3K27ac, ChIP-seq
GSM8792003 WCM677_1, H3K27me3, ChIP-seq
GSM8792004 WCM677_3, H3K27ac, ChIP-seq
GSM8792005 WCM677_3, H3K27me3, ChIP-seq
GSM8792006 WCM90_Z21, H3K27ac, ChIP-seq
GSM8792007 WCM90_Z21, H3K27me3, ChIP-seq

Genome browser session
(e.g. UCSC)

NA

Methodology

Replicates	Replication was not performed due to the use of patient samples.
Sequencing depth	Each ChIP-seq sample was sequenced to a depth of 20 million single-end reads. Each CUT&Tag sample was sequenced to a depth of 20 million paired-end reads.
Antibodies	ChIP-seq studies were performed using antibodies against H3K27me3 (cell signaling technology; cat. no. 9733S) or H3K27ac (Diagenode; cat. no. C15410196).
Peak calling parameters	Peak calling was conducted employing MACS2 with a genome size of 2.7e9 and the standard FDR threshold of 0.01. For H3K27me3 data, the flag "--broad" was used.
Data quality	Peaks were called at an FDR set to less than 0.01.
Software	Sequencing reads were mapped to hg38 human genome reference using bowtie2 version 2.2.5. Peak calling was conducted employing MACS2. A union set of peaks was generated using BEDTools. For each sample, fragment counts within each histone peak were extracted using the getCounts function from chromVAR, followed by variance-stabilizing transformation (vst) normalization using DESeq2.
